# Partial isolated rupture of the popliteus tendon in a professional soccer player: a case report

**DOI:** 10.1186/1758-2555-1-18

**Published:** 2009-07-31

**Authors:** Pier Paolo Mariani, Fabrizio Margheritini

**Affiliations:** 1 Department of Health Sciences, Unit of Sports Traumatology, Piazza L. de Bosis 6, University of Rome "Foro Italico", I-00135 Rome, Italy

## Abstract

The complete isolated rupture of the popliteus tendon has been described as a rare injury and this report describes the case of a 31-year-old soccer player who sustained a partial rupture of the popliteus tendon during a game. The injury was suspected clinically and at MRI but confirmed only by the arthroscopic examination. The treatment consisted in open debridment with no tendon repair or augmentation. Seven weeks post-operation the patient was symptom-free and returned to competitive professional soccer at the same preinjury level. The clinical and arthroscopic findings of the case reported suggest a possible overuse disease with degenerative expression.

## Introduction

The popliteus muscle functions as a dynamic internal rotator of the tibia. For this reason rupture of the popliteus muscle is usually associated with acute or chronic posterolateral instability of the knee. The isolated rupture of the popliteus tendon is a very rare injury and it has been rarely reported in the literature as a cause of knee hemarthrosis and functional disabilities. While most of the reported cases deal with tendon avulsions [1-6] or intra-substance ruptures [7,8], partial rupture has never been reported previously. The purpose of this article is to report one case of partial rupture of the popliteus tendon in a professional soccer player treated by debridment and to discuss the pathological findings of this lesion that could suggest an overuse disease of the tendon in a athlete.

## Case presentation

**Figure 1:**
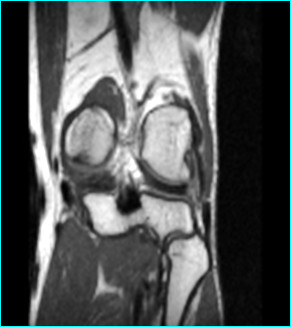
**Coronal T1 weighted image showing a mild inflammation within the popliteus tendon course, but not clear signs of tear**.

**Figure 2:**
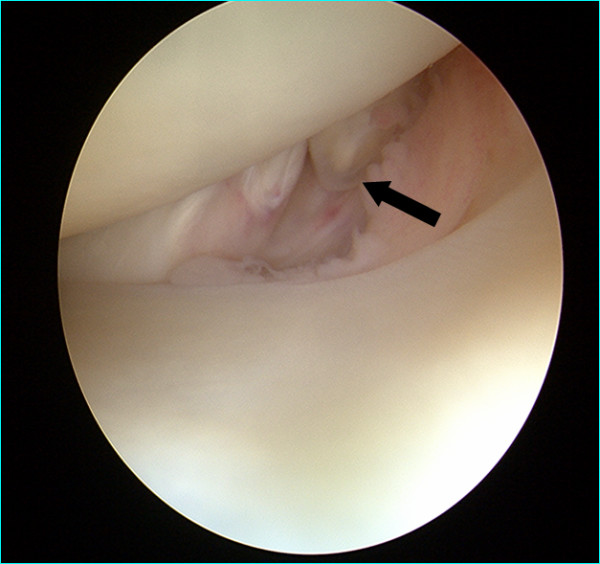
**Arthroscopic view of the partial rupture with the knee in Cabot's position**.

**Figure 3:**
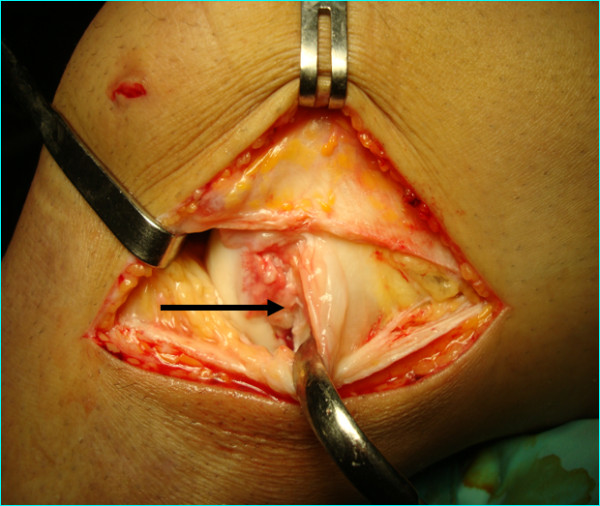
**View of the popliteus tendon (arrow) after its open exposure**. With the knee flexed at 90 degrees, a vertical arthrotomy has been carried out anteriorly to the popliteus tendon. The capsula and the lateral collateral ligament are retracted by the hook. Note the presence of inflammatory tissue around the femoral insertion of popliteus tendon.

A 31-year-old professional first division goalkeeper injured his right knee hitting the ground with the lateral side of the knee after making a save during a game. No previous similar injuries were sustained by the player, while he wasn't able to identify the exact moment of the trauma. He referred immediately only minimal discomfort and no apparent effusion and he continued to play on. After 2 weeks, the discomfort progressed despite physiotherapy. An orthopaedic referral was made for persistent pain located at knee lateral aspect only during his sports activity. The first orthopaedic surgeon made a clinical diagnosis of a lateral meniscal tear and requested an MRI examination that was judged negative. At that time the patient came to our institution without any further treatment carried on. Clinical examination revealed absence of effusion and non-specific lateral joint-line tenderness with full extension and flexion. It was possible to elicit sharp pain palpating the popliteus tendon at femoral groove. The suspicion of other diseases, such as popliteus tendon snapping or popliteomeniscal fascicle tear were ruled out with the figure-four test and the Cabot test that were both negative. There were no further signs of meniscal pathology. The range of motion was unremarkable, with no instability to varus or valgus stress at 0° or 30° of knee flexion. Furthermore non signs of posterolateral rotatory instability were possible to elicit. MRI showed a mild inflammation around the popliteus tendon with no specific sign of rupture, (Fig. 1). The patient underwent a diagnostic arthroscopy approximately four weeks after the trauma due to the lack of results with a prior conservative treatment. At the time of surgery, a complete examination under anaesthesia showed normal knee laxity under manual laxity tests with no signs of rotatory instability. Arthroscopic examination was carried out with a standard anterolateral and anteromedial portals and revealed a partial popliteus tendon rupture just above the hiatus, (Fig. 2). The anterior fibers of the tendon were torn and a small stump was visible. The examination of the hiatus did not show the presence of a distal stump. There was no meniscal or cruciate pathologic conditions. In order to check the mechanical efficiency of the tendon and with the plan of performing an augmentation of the tendon if necessary, an open approach was performed that confirmed the presence of a partial tendon tear. The tendon was tested with an external rotatory force applied to the tibia [9]. Because of absence of increased laxity or tendon insufficiency, verified under direct view with internal and external rotation of the tibia and pulling with a clamp the tendon, we performed only an open debridement of the stump, (Fig. 3), and the inflammatory tissue around the tendon was gently removed. Post-operatively the patient was allowed full weight bearing wearing a brace for four weeks time. The patient was symptom free 7 weeks post-op, playing competitive professional soccer.

## Discussion

The anatomy of the popliteus tendon is well known. The popliteus muscle arises from the proximal posterior surface of the tibia and has insertions into the posterior portion of the lateral meniscus and the femur, both deep and anterior to the lateral collateral ligament. The femoral insertion has a crescent shape, with the superior aspect being concave [10]. The main tendon of the popliteus muscle consists of anterior and posterior fibers. Electromyographic recordings have shown that the muscle acts as a prime medial rotator of the knee joint and in the crouching position is the only muscle in a position to prevent the femoral condyles from gliding forward on the tibia [11]. Some anatomical variants of the popliteus tendon have been described without clinical relevance[12,13].

Isolated ruptures of the popliteus tendon are rare and few cases are described in the orthopaedic literature. The lesion is more frequently reported as femoral avulsion and only in two cases [6,8] a complete intrasubstance tear has been identified. Mechanism injury is often misleading and unclear and different mechanisms have been described. Lesions have been reported occurring by a non contact external rotational mechanism: a sudden external rotation to a partially flexed knee [1,4,5], a forced external rotation with a varus force application in some cases, or a forced external rotation with femur fixed [2] have been described. In our case, no specific injury was recalled by the athlete and probably, also considering the type of tear, an overuse or degenerative mechanism could be considered as responsible of the partial tear.

In the avulsion type, an acute haemarthrosis without laxity signs and pain on lateral aspect of knee should lead to suspicion of such a lesion. In the other cases without avulsion, as in our patients, the findings on physical examination are subtle and reveal only discomfort just over the popliteus tendon in an otherwise stable knee. So, the diagnosis is easily overlooked. The diagnosis must be verified by an arthroscopic examination because also the MRI, usually helpful, can be in this pathology unclear. Arthroscopy should be considered an additional step in the process of information gathering [14] and in case of isolated lesion as reported by previous authors an arthroscopic debridement can be carried out at the same time.

Treatment of isolated rupture of the popliteus tendon is not well defined. Despite the relatively small number of cases presented, different treatments have been reported in literature. Rose and Parisien [1] presented in the late 80's the first known case of isolated rupture of the popliteus tendon after an indirect trauma, treated with an open repair and full recovery. Gruel [3], a few years later, presented two cases of rupture of the tendon with an associated bone avulsion treated with an arthroscopic debridment and a bone removal without repair of the torn tendon followed by a complete recovery at two years follow-up. Burstein and Fischer [4] presented the same outcome after a similar lesion and treatment in a professional football player who sustained a complete rupture. The authors performed a diagnostic arthroscopy and no further surgical treatment was performed. Westrich et al. [7] presented a case of isolated rupture repaired using two suture anchors, with a complete recover and no signs of lateral/posterolateral instability at its longest follow-up. In this last case it was also associated a partial rupture of the ACL. It is interesting to note that the case of isolated rupture of popliteus reported by Westrich et al. [7] was the only case that presented a subtle, but clinically detectable, preoperative posterolateral laxity. Mirkopulos et al [5] reported the case of a young basketball player who underwent surgical reattachment of the avulsed portion of the popliteus tendon using metallic screw and washer.

More recently, Conroy et al. [8] presented a case of isolated complete rupture in a professional soccer player, with a intra-susbtance tear that was treated by arthroscopic debridment of the stumps with a final return, 6 weeks later, to competitive soccer level. Our case is the first reported case to our knowledge of partial popliteus tendon lesion. On the basis of clinical history this case should be distinguished from the popliteus acute tear and the symptoms were related to mechanical impingement of the torn fibers with the lateral meniscus. Also the arthroscopic findings suggest an overuse disease of the tendon. They are quite similar to that reported by Conroy et al. [8]. For the absence of hemarthrosis and of clear signs of acute injury we did not perform the repair of the tendon. We have only carried out the debridement of the fibers responsible of local symptoms and we have checked the residual mechanical efficiency of the tendon.

The clinical result achieved in this first division professional goalkeeper underlined the uselessness of tendon suture or any posterolateral ligament reinforcement in case of isolated partial rupture of the tendon, with no rotational instability, with an almost complete recovery at medium term follow-up.

## Consent

Written informed consent was obtained from the patient for publication of this case report and accompanying images. A copy of the written consent is available for review by the Editor-in-Chief of this journal

## Competing interests

The authors declare that they have no competing interests.

## Authors' contributions

Both MPP and FM conceived of the study, and participated in its design and coordination. All authors read and approved the final manuscript.
